# Local Control After Adjuvant Radiosurgery for Spinal Metastasis Treated With Decompression and Posterior Segmental Stabilization: A Comparison Between Carbon Fiber/Polyetheretherketone-Based and Metallic Implants

**DOI:** 10.1016/j.adro.2025.101806

**Published:** 2025-06-10

**Authors:** Romulo A. Andrade-Almeida, Francisco Call-Orellana, Juan P. Zuluaga-Garcia, Esteban Ramirez-Ferrer, Gil Kimchi, Brian S. De, Alexandre B. Guimaraes, Christopher A. Alvarez-Breckenridge, Jing Li, Amol J. Ghia, Laurence Rhines, Martin C. Tom, Chenyang Wang, Thomas H. Beckham, Behrang Amini, Robert Y. North, Claudio E. Tatsui

**Affiliations:** a Department of Neurosurgery, The University of Texas MD Anderson Cancer Center, Houston, Texas, USA; b Department of CNS Radiation Oncology, The University of Texas MD Anderson Cancer Center, Houston, Texas, USA; c Department of Neurosurgery, Hospital das Clinicas da Faculdade de Medicina da USP, Sao Paulo, Sao Paulo, Brazil; d Department of Diagnostic Imaging, The University of Texas MD Anderson Cancer Center, Houston, Texas, USA

## Abstract

**Purpose:**

Carbon fiber-reinforced polyetheretherketone (CFRP) spinal implants are gaining popularity in the surgical management of spinal metastasis because of their physical and radiographic properties, which facilitate adjuvant radiation planning and tumor surveillance. Their impact on clinical outcomes is still under investigation. We evaluated the role of hardware material (CFRP vs titanium) in local control and hardware durability in metastatic cases receiving decompressive surgery and adjuvant spinal stereotactic radiosurgery (SSRS).

**Methods and Materials:**

This single-institution, retrospective cohort study was approved by the local institutional review board. Patients who underwent decompressive surgery with posterior segmental instrumentation followed by treatment with SSRS for metastatic spine disease were included. Exclusion criteria were as follows: (1) cervical implants, (2) mixed-type hardware, (3) SSRS greater than 60 days after surgery, and (4) less than 3 months magnetic resonance imaging follow-up. Only tumor progression occurring inside or at the margins of the irradiated field was considered.

**Results:**

Eighty-three spinal segments (55 titanium, 28 CFRP; from 82 patients) were evaluated. Except for the number of radiation fractions, proportion of single-fraction 24 Gy cases, and radiation equivalent dose in 2-Gy fractions, no significant differences were found between groups. The median follow-up time was 14.5 months (range, 3.0-70.4 months). Sixteen local progressions were identified, with 15 in the titanium group (*P* = .009). Using death as a competing factor, local progression-free survival was longer in the CFRP group (HR, 0.127; 95% CI, 0.017-0.945; *P* = .044). The median time to progression was 9.27 months (IQR, 4.5-15.65 months). Higher equivalent dose in 2-Gy fractions was the only variable associated with local tumor control in both univariate and multivariate analyses (*P* = .025 and *P* = .019, respectively). The titanium cohort experienced 4 hardware adverse events, whereas the CFRP group had 2 adverse events (*P* > .05).

**Conclusions:**

CFRP implants were associated with lower rates of local progression in crude analyses, but did not reach statistical significance in multivariable models. No differences in hardware durability were identified.

## Introduction

Spinal metastases are relatively frequent in patients with cancer, with a reported incidence ranging from 20% to 70%, with 10% of these cases developing symptomatic metastasis (ie, presenting with pain and/or incapacity).^1^, ^2^, ^3^, ^4^ The number of patients affected with this condition is expected to rise because of the increasing prevalence of people living with cancer as the newer and more effective targeted therapies, immunotherapies, and systemic therapies increase the survival rates across various tumor histologies.^1^^,^^5^ The management of spinal metastasis with high-grade spinal cord compression frequently involves surgery for tumor resection, spinal stabilization, and adjuvant spinal stereotactic radiosurgery (SSRS).^3^^,^^6^ Renal, hepatocellular, colon, non-small cell lung carcinomas, sarcoma, and melanoma are examples of radioresistant tumors often benefiting from dose escalation with SSRS.^7^ Stabilization is obtained through hardware implantation, and the posterior approach with placement of pedicle screws and rod constructs is the most frequently used method.^8^ However, spinal hardware is associated with several potential problems, including radiographic artifacts on surveillance imaging resulting in impaired assessment of local control, more challenging radiation planning, and mechanical failure.^9^^,^^10^

Currently, most spinal implants are made of metals (eg, titanium), which have been shown to produce a greater amount of artifacts on magnetic resonance imaging (MRI) and computed tomography (CT) scans when compared to carbon fiber-reinforced polyetheretherketone (CFRP), a carbon-based material with radiolucent properties.^9^^,^^11^^,^^12^ CFRP also interacts less with radiation therapy, particularly protons, when compared to titanium, resulting in reduced undesired backscatter and shielding effects.^13^^,^^14^ Some authors have hypothesized that this combination of inferior imaging visualization and difficult radiation planning/delivery may result in worse local outcomes in patients with spinal tumors.^9^^,^^15^ Data on oncological outcomes, however, are scarce throughout the literature, and the papers studying this topic often include a heterogeneous population of patients. Nevertheless, data on CFRP biomechanics support that these constructs have comparable strength to their metallic counterparts.^16^^,^^17^

We report our institutional experience with surgical decompression and spinal stabilization of patients with high-grade spinal cord compression from metastatic disease treated with titanium or CFRP implants followed by SSRS. The primary objectives were to evaluate differences in local control and durability of the spinal reconstruction for CFRP versus titanium.

## Methods and Materials

### Study design and population

We conducted a retrospective cohort study on consecutive patients who had both spinal surgery and radiation treatment between October 2007 and December 2023 at our institution. Only patients who met all the following inclusion criteria were evaluated: (1) decompression and placement of posterior segmental instrumentation (titanium or CFRP), and (2) spinal metastasis treated with SSRS. Cases were excluded if: (1) utilization of mixed hardware material (eg, CFRP screws with titanium rods, or screws of different material in the same construct); (2) construct had a cervical extension; (3) patient received SSRS ≥60 days after surgery; or (4) patient had <3 months of imaging follow-up with MRI.

### Ethics

The execution of this study complied with a protocol approved by the institutional review board.

### Interventions

In general, patients were selected for surgery if presenting with a symptomatic or critical (Bilsky grades 1c, 2, and 3)^18^ spinal cord compression secondary to a tumor. The procedures were all single-stage posterior transpedicular circumferential dural decompression and pedicle screw spinal stabilization. More extensive resections included a vertebral body reconstruction using either polymethyl methacrylate or a nonmetallic cage. Titanium cages were only allowed in the titanium cohort. All CFRP cases were done using screws and rods from icotec. These screws are manufactured with a titanium polyaxial screw head and a CFRP shaft coated with porous titanium; the rods are made from pure CFRP and available in multiple precontoured curvatures and lengths and cannot be bent. Durable local tumor control was sought by delivering SSRS as soon as wound healing status and patient condition allowed. SSRS plans were made in either the Pinnacle (Philips) or Raystation (RaySearch Laboratories) software, typically using the preoperative and postoperative MRI coregistered with a postoperative, preradiation simulation CT scan and CT myelogram. After discharge, patients are usually assessed with 1.5 T or 3.0 T control MRI every 3 months for the first 1 to 2 years, followed by imaging every 6 months.

### Data and analyses

Data for eligible cases were retrieved from electronic medical records. Variables of interest included demographics, dates of birth and death or last follow-up, date of surgery, need for reoperation, reason for reoperation, date and doses of radiation treatments, date of local progression or last MRI, date of hardware failure detection or last CT scan, primary histology, radiosensitivity profile, vertebral levels affected by tumor, construct length, vertebral levels instrumented, number of screws used, modality of vertebral body reconstruction, hardware material, and preoperative Karnofsky Performance Scale.

Hardware durability was assessed based on the review of all postoperative CT scans. Date of mechanical failure was calculated from the date of surgery and the date of detection of any of the following image features: presence of haloing around the whole length of the pedicle screws shaft, subsidence of the pedicle screws or the interbody reconstruction device (polymethyl methacrylate block or cage), and breakage of pedicle screws or rods. Broken instrumentation that required revision surgery to replace components or add more points of fixation to stabilize the spine were analyzed separately.

MRI reports done by expert neuroradiologists and musculoskeletal radiologists comparing the first posttreatment scan to subsequent studies were used to define local progression. Appearance of new enhancing mass, increase of more than 25% of any residual disease, or increase in the Bilsky score within the surgical site were used as parameters to define progression. The interval from the last day of SSRS to the MRI confirming local progression, or the last MRI (if no progression), was calculated. Local progression was classified as in-field (completely inside the gross total volume [GTV]) or marginal (within the clinical target volume [CTV]).

For statistical analyses, IBM SPSS Statistics v. 24 and R Studio were used, with an alpha level of 5% set for statistical significance. Comparisons between means were conducted using either the Student’s or Welch’s *t* test, depending on the result of Levene’s test for equality of variances. Whenever a violation of the normality assumption was detected, the comparison between medians was calculated by a nonparametric test. χ^2^ tests were used to compare categorical variables, with Pearson’s or Fisher exact test selected based on their applicability. Time-to-event variables were analyzed using Firth’s penalized Cox proportional hazards analysis and a Kaplan-Meier curve with respective log-rank test comparing local progression probabilities over time between titanium and CFRP; the final model was determined based on clinical relevance, and for variables that did not reach statistical significance, the Hosmer and Lemeshow criteria were used for final multivariate model selection. A competing risk analysis was conducted to evaluate the cumulative incidence of 2 mutually exclusive variables (local progression vs death). The cumulative incidence functions were estimated for each hardware type to account for the competing nature of these events. The Fine and Gray test was used to compare the cumulative incidence functions between the groups, assessing whether there was a statistically significant difference in the incidence of events over time. Accounting for these events allows for a more accurate estimation of local tumor progression likelihood.^19^ The penalized maximum likelihood method (Firth's correction) was used to explore potential variables related to hardware failure in a parsimonious multivariate analysis. This model was selected because of the small sample size of hardware failure cases in our cohort, helping to mitigate bias associated with small sample sizes and sparse data. The final variables included in the model were the number of screws, the need for anterior column repair, and the type of hardware.^20^ This method was specifically chosen to handle issues of perfect separation and small sample sizes within certain predictor categories. Penalizing the likelihood function reduces bias and allows for more reliable estimates of odds ratios and valid inference.

## Results

**Figure 1:**
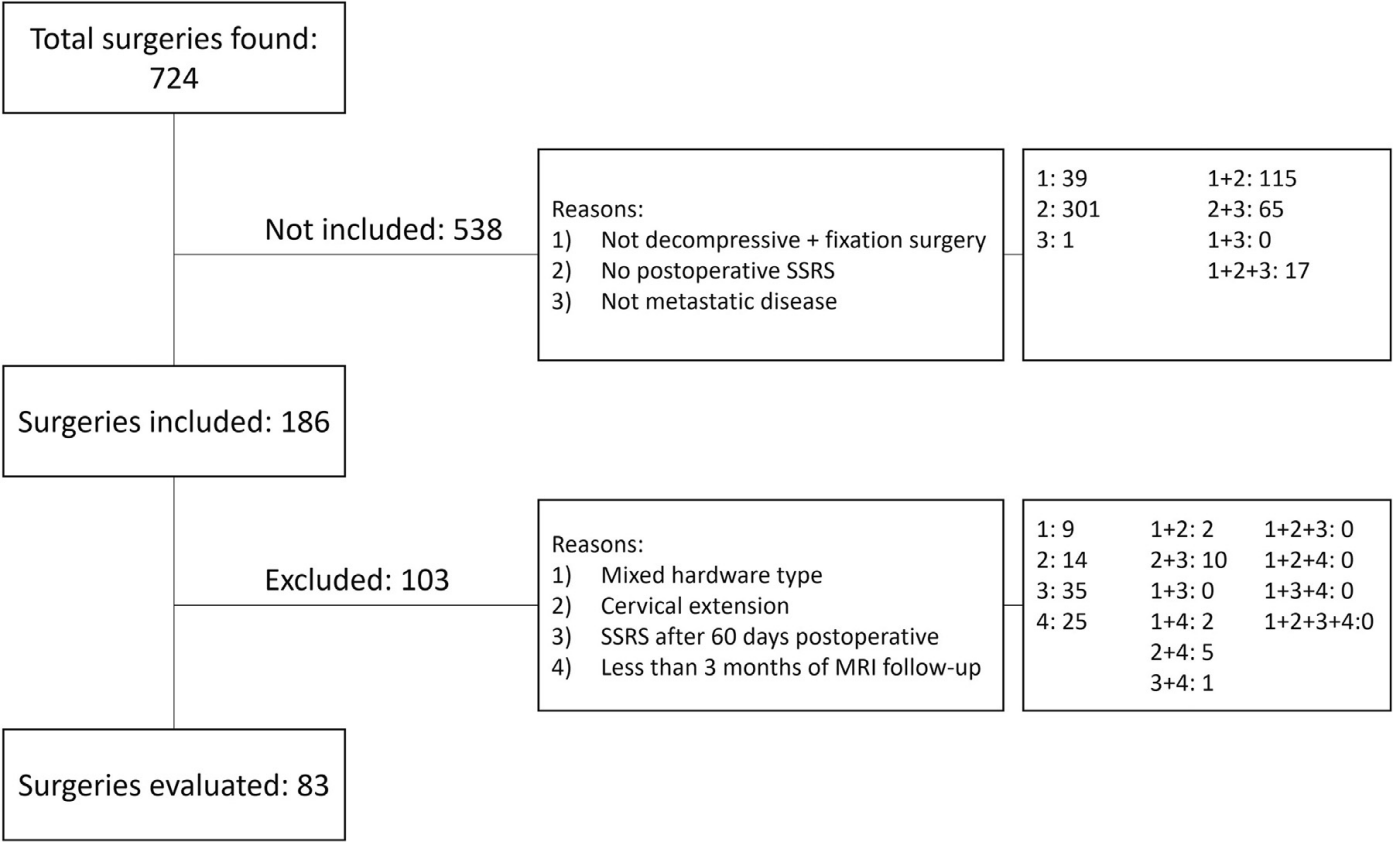
Flowchart for the application of inclusion and exclusion criteria for case selection, with results for each step. *Abbreviations:* MRI = magnetic resonance imaging; SSRS = spinal stereotactic radiosurgery.

Seven hundred twenty-four cases were identified from our institutional database. From these, 83 cases (from 82 patients) were included after applying the predefined inclusion and exclusion criteria and divided between the titanium (n = 55) and CFRP (n = 28) groups (Fig. 1). Comparisons between these 2 cohorts across multiple demographic, clinical, surgical, and treatment variables did not show any baseline difference except for the mean equivalent dose in 2-Gy fractions (EQD2), number of SSRS fractions, and proportion of patients receiving single-fraction 24 Gy. No significant differences were found for the remaining categories (Table 1). Considering breast, prostate, small cell lung cancer, thyroid, and head and neck squamous cell carcinoma as radiosensitive tumors, there was not a significant difference in radiosensitive and nonradiosensitive (including radioresistant and unclear resistance) proportion between groups.^7^^,^^21^

**Table 1:** Comparison between demographic data of titanium vs CFRP groups

Variable	Titanium (n = 55)	CFRP (n = 28)	*P* value
Sex, n (%)			
Female	19 (34.5)	13 (46.4)	.293*
Male	36 (65.5)	15 (53.6)	
Age at surgery, y, median (IQR)	60.5 (12.4)	57.9 (15.0)	.093†
Revision case, n (%)			
Yes	4 (7.3)	1 (3.6)	.658‡
No	51 (92.7)	27 (96.4)	
Number of vertebrae affected, median (IQR)	1.2 (0.5)	1.3 (1.0)	.342†
Construct length, vertebral levels, median (IQR)	5 (0.0)	5 (0.0)	.826†
Transitional segment, n (%)			
Yes	15 (27.3)	7 (25.0)	.824*
No	40 (72.7)	21 (75.0)	
Screw quantity, median (IQR)	8 (1.0)	8 (0.5)	.948†
Prior radiation to the same segment, n (%)			
Yes	6 (10.9)	3 (10.7)	1.000‡
No	49 (89.1)	25 (89.3)	
SSRS EQD2 (α/β 10), Gy, median (IQR)	42.8 (25.3)	68.0 (0.0)	.003†^,^§
SSRS fractionation, n (%)			
Single fraction	32 (58.2)	24 (85.7)	.013‡^,^§
Multi fraction	23 (41.8)	4 (14.3)	
SSRS fractionation, median (IQR)	1 (2.0)	1 (0.0)	.012†^,^§
Received 24 Gy/1 fraction, n (%)			
Yes	23 (41.8)	22 (78.6)	.001*^,^§
No	32 (58.2)	6 (21.4)	
KPS at surgery, median (IQR)║	90 (10.0)	90 (10.0)	.448†
Histology			
RCC	21 (38.2)	13 (46.4)	-
Lung adenocarcinoma	4 (7.3)	1 (3.6)	-
Breast IDC	3 (5.5)	0 (0.0)	-
Colorectal	3 (5.5)	1 (3.6)	-
Prostate adenocarcinoma	3 (5.5)	0 (0.0)	-
Unknown primary	3 (5.5)	1 (3.6)	-
HCC	2 (3.6)	0 (0.0)	-
Lung SCC	2 (3.6)	0 (0.0)	-
Urothelial carcinoma	2 (3.6)	0 (0.0)	-
Leiomyosarcoma	0 (0.0)	4 (14.3)	-
Paraganglioma	0 (0.0)	2 (7.1)	-
Other¶	12 (21.8)	6 (21.4)	-

*Abbreviations:* CFRP = carbon fiber-reinforced polyetheretherketone; cGy = centigray; EQD2 = equivalent dose in 2-Gy fractions; HCC = hepatocellular carcinoma; IDC = invasive ductal carcinoma; IQR = interquartile range; KPS = Karnofsky performance status; RCC = renal cell carcinoma; SCC = squamous cell carcinoma; SSRS = spinal stereotactic radiosurgery.

⁎ *P* value from Pearson χ^2^.

† *P* value from the Mann-Whitney U test.

‡ *P* value from Fisher exact test.

§ Significant *P* values.

║ KPS data were available for 32 individual cases in the titanium group, and 21 individual cases in the CFRP group.

¶ Corresponds to histology types with one case each. In the titanium group, it included adenoid cystic adenocarcinoma, cholangiocarcinoma, epitheloid fibrosarcoma, follicular thyroid carcinoma, head and neck SCC, melanoma, olfactory neuroblastoma, papillary thyroid carcinoma, poorly differentiated esophageal carcinoma, prostate SCC, salivary gland carcinoma, and small cell lung carcinoma. In the CFRP group, it included adrenal cortical carcinoma, cholangiocarcinoma, colorectal, epitheloid fibrosarcoma, head and neck SCC, pericytoma, and poorly differentiated thyroid carcinoma.

### Local tumor control analyses

**Figure 2:**
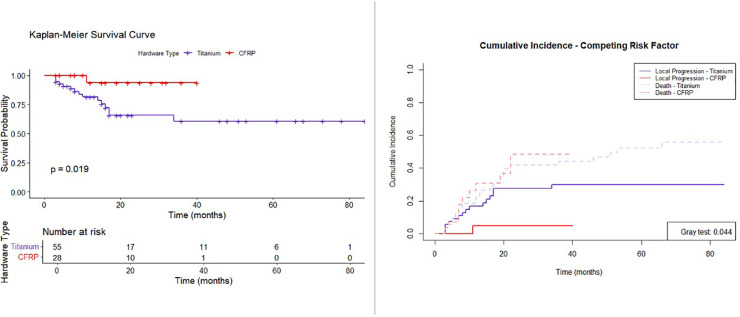
Survival and cumulative incidence curves for local tumor control. The left panel shows the Kaplan-Meier survival curve comparing progression-free survival probabilities over time between 2 hardware types: titanium (purple) and carbon fiber-reinforced polyetheretherketone (CFRP) (red), with a *P* value indicating the significance of the difference. The right panel displays a competing risk analysis, illustrating the cumulative incidence of local progression and death for both hardware types, with a Gray test *P* value for the comparison.

Patients were followed with serial MRI for a median time of 14.5 months (interquartile range [IQR], 22.9 months; range, 3.0-70.4 months), with a median follow-up time of 16.8 months in the titanium group and 12.4 months in the CFRP group (*P* = .303). Overall survival analysis did not show a significant difference between groups (log-rank, *P* = .542). Most local progressions (n = 15) occurred in the titanium group (only one local progression in the CFRP group), with a statistically significant difference in proportion of progression (titanium, 27.3% and CFRP, 3.6%; *P* = .009). The median time to progression was 9.27 months (IQR, 4.5-15.65 months). The Kaplan-Meier survival curve (Fig. 2, left panel) indicates a significant difference in survival probabilities over time between the titanium and CFRP groups (log-rank test, *P* = .019), suggesting that the CFRP hardware may influence survival outcomes over time. The results of the competing risk analysis comparing the cumulative incidence of local progression and death for both hardware types are presented in Fig. 2, right panel. The Gray test *P* value reveals a statistically significant difference, indicating that CFRP is associated with a lower risk of local progression compared to titanium (HR, 0.127; 95% CI, 0.017-0.945). Despite the difference in risk of local progression, the Cox regression models (Table 2) showed that hardware type was not significantly associated with this outcome, both in univariate (HR, 0.14; *P* = .060) and multivariate (HR, 0.20; *P* = .110) analyses. This discrepancy suggests that, although CFRP may have a protective effect in simpler analyses, the influence of confounding variables may reduce its apparent impact when adjusted for in the Cox model. Similarly, vertebral body reconstruction, prior radiation, and nonradiosensitive histology did not significantly affect the risk of local progression. However, the fractionation scheme was significantly associated with an increased risk of progression in the univariate analysis (HR, 4.80; *P* = .003), suggesting that multiple fractions might lead to worse outcomes. Conversely, we have found an association between higher EQD2 (α/β = 10) and a reduced risk of local progression, with a significant HR of 0.95 (*P* = .025) in the univariate analysis, and of 0.94 (*P* = .019) in the multivariate model. These results indicate that although hardware type may play a role, radiation dose and fractionation scheme are the key factors influencing the risk of local progression.

**Table 2:** Cox proportional hazards analysis of factors influencing local progression

Variable	Categories	Frequency	HR (univariate)	HR (multivariate)
Hardware	Titanium	55 (66.3)	0.14 (0.02-1.09, *P* = .060)	0.2 (0.02-1.49, *P* = .110)
	CFRP	28 (33.7)		
Vertebral body reconstruction	No	53 (63.9)	1.10 (0.41-2.97, *P* = .850)	0.51 (0.17-1.58, *P* = .190)
	Yes	30 (36.1)		
Prior Radiation	No	74 (89.2)	0.90 (0.12-6.93, *P* = .922)	0.66 (0.06-4.36, *P* = .557)
	Yes	9 (10.8)		
Nonradiosensitive histology	No	69 (83.1)	0.70 (0.16-3.08, *P* = .636)	0.24 (0.05-1.29, *P* = .098)
	Yes	14 (16.9)		
Fractionation scheme	Single	56 (67.5)	4.80 (1.71-13.48, *P* = .003)*	-
	Multiple	27 (32.5)		
GTV volume, cc	Mean (SD)	76.5 (68.8)	1.00 (1.00-1.01, *P* = .689)	1.00 (0.99-1.00, *P* = .497)
EQD2	Mean (SD)	56.4 (12.7)	0.95 (0.91-0.99, *P* = .025)*	0.94 (0.89-0.98, *P* = .019)*
Minimum GTV dose, EQD2	Mean (SD)	16.3(6.3)	0.98 (0.89-1.07, *P* = .661)	-

*Abbreviations:* cc = cubic centimeter; CFRP = carbon fiber-reinforced polyetheretherketone; EQD2 = equivalent dose in 2-Gy fractions; GTV = gross total volume; HR = hazard ratio; SD = standard deviation.

⁎ Significant *P* values.

**Figure 3:**
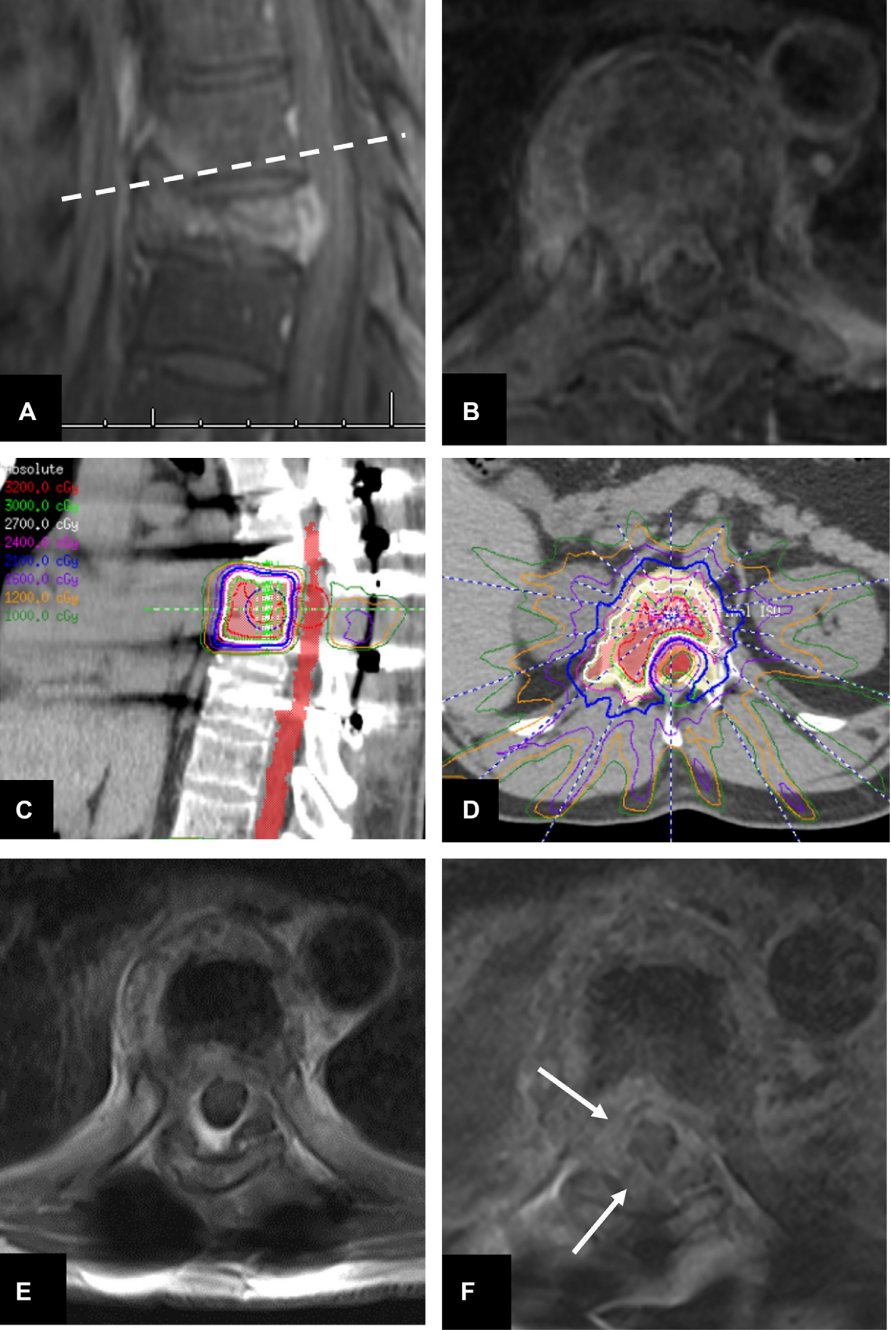
Images from a 50-year-old male with a history of colon cancer metastatic disease to T8 and L1 presenting with progressive bilateral lower extremity weakness during a course of conventional radiation therapy. (A) Postcontrast T1-weighted magnetic resonance imaging showed vertebral compression fracture of T8 with an epidural tumor component (B), causing spinal cord compression. The dashed line corresponds to the level of the axial cuts shown in (E) and (F). Patient underwent partial T8 vertebrectomy and T6 to T10 instrumentation with titanium implants—his neurologic function improved after surgery. (C) and (D), 2700 cGy was delivered in 3 fractions to the same level, from endplate to endplate. (E) Surveillance postcontrast T1-weighted magnetic resonance imaging demonstrating a stable tumor; however, 14 months later, progressive disease (white arrows) was seen in the margins of the irradiated field. Because of the overspread progressive disease, the patient was discharged to hospice care, and died 1 month later.

From the 15 cases experiencing local progression in the titanium group, 11 (73.3%) occurred within the GTV (in-field), whereas the other 4 (26.7%) cases occurred at the margins of this area. The recurrence case in the CFRP group occurred within the GTV. Individual data for these recurrent cases are presented in Table 3, with an illustrative case shown in Fig. 3.

**Table 3:** Individual data for cases that had local tumor progression

Progression case ID #	Group	Histology	SSRS total dose, cGy/fractions	Time to progression, mos	Area of progression
1	Titanium	RCC	2000/1	32.9	In-field
2	Titanium	Lung SCC	2700/3	2.2	In-field
3	Titanium	Fibrosarcoma	2700/3	15.3	In-field
4	Titanium	Colon adenocarcinoma	2700/3	15.7	In-field
5	Titanium	HCC	2700/3	3.7	In-field
6	Titanium	Unknown primary	2700/3	9	In-field
7	Titanium	RCC	2700/3	9	Margin
8	Titanium	Colon adenocarcinoma	2700/3	4	In-field
9	Titanium	Unknown primary	2400/1	7	Margin
10	Titanium	Unknown primary	2700/3	14	Margin
11	Titanium	RCC	2400/1	5.3	In-field
12	Titanium	RCC	2700/3	15	In-field
13	Titanium	Urothelial carcinoma	2400/1	3	Margin
14	Titanium	Prostate adenocarcinoma	2700/3	3	In-field
15	Titanium	Prostate adenocarcinoma	1800/1	14	In-field
16	CFRP	RCC	2400/1	1	In-field

*Abbreviations:* CFRP = carbon fiber-reinforced polyetheretherketone; cGy = centigray; HCC = hepatocellular carcinoma; RCC = renal cell carcinoma; SCC = squamous cell carcinoma; SSRS = spinal stereotactic radiosurgery.

### Durability of spinal hardware

Data were available for 81 cases (2 cases did not have follow-up CT scan), which had their constructs followed for a median time of 16 months (IQR, 21 months; range, 3-84 months). There were a total of 6 mechanical failures detected, divided between the titanium (n = 4/53, 7.5%) and CFRP (n = 2/28, 7.1%) groups, without significant difference (*P* = 1.000); Mechanical hardware failure-free survival was not different between groups (log-rank test, *P* = .47). The titanium cohort presented with cage subsidence (n = 2), screw subsidence (n = 1), and bilateral broken screws in the superior portion of the construct (n = 1). In contrast, the CFRP group had one case of bilateral screw breakage at the inferior end of the construct, and one case of complete haloing around a screw on the upper part of the construct. There was no difference between groups in the proportion of cases needing revision surgery to address hardware-related complications (titanium, 1 [1.8%], CFRP, 2 [7.1%]; *P* = .262). Univariate analyses were performed to determine risk factors for hardware failure. Variables tested included age (*P* = .807), sex (*P* = .796), reoperation case (*P* = 1.000), hardware material (*P* = 1.000), construct length (*P* = .161), vertebral body reconstruction (*P* = .232), cement augmentation (*P* = .404), and fixation of a transitional segment (*P* = .182), and short-segment reconstructions (one level above and one level below to vertebra with tumor) (*P* = .624). In the multivariate analysis (Firth’s penalized likelihood approach), considering all the variables investigated in univariate analysis, the higher number of screws was significantly associated with a reduced likelihood of hardware failure (*P* = .007). Additionally, anterior column repair was associated with an increased risk of hardware failure (*P* = .035). The type of hardware did not significantly impact hardware failure (*P* = .613).

We observed a trend toward a correlation between short constructs and the need for revision surgery for broken hardware (*P* = .052); the same tendency was not seen when comparing the material of the spinal hardware (*P* = .111). Of note, no rod fractures were seen in this cohort.

## Discussion

The development and integration of SSRS in the management of spinal metastasis allows effective treatment of radioresistant and recurrent neoplasms.^4^ In our institution, postoperative SSRS planning includes the utilization of the preoperative MRI or CT scans for target delineation, a postoperative CT myelogram for spinal cord definition, and a postoperative MRI fused to the treatment planning CT scan to delineate the areas to be irradiated and organs at risk. For adequate treatment planning, all images must be of high quality and allow a clear delineation of structures, especially when the area to be irradiated is in close contact with one or more organs at risk. The contouring of the tumor and spinal cord is more complex in the postoperative setting because of the presence of fluid collections, tissue edema, and metallic implants that create imaging artifacts.^9^^,^^22^ Surveillance imaging is also affected by these artifacts, and may cause a delay in local progression detection.^11^

The increasing use of CFRP as an alternative to titanium in the manufacturing of spine implants is primarily driven by growing evidence of its advantages over the metallic counterpart, particularly enhancing the quality of postoperative imaging, which facilitates the radiation planning.^13^^,^^23^ CFRP produces fewer artifacts on CT and MRI scans, improving tumor surveillance^9^^,^^12^^,^^13^^,^^24^ and treatment delivery when particle therapy is used, as it offers less backscatter and shielding effects than titanium, which may enhance target coverage homogeneity.^9^^,^^13^^,^^14^^,^^25^, ^26^, ^27^, ^28^ The presence of tantalum in the tip of CFRP screws facilitate intraoperative visualization and have been described as fiducial markers for intrafraction motion tracking in patients receiving SSRS.^29^ Regarding its biomechanical properties, CFRP constructs offer reliable and durable stabilization, with bending stiffness, fatigue strength, maximum axial load, maximum compression, and screw loosening resistance comparable or superior to titanium counterparts.^16^^,^^30^^,^^31^ However, studies investigating the translation of these properties in terms of hardware durability, fusion rates, and functional outcomes remain limited.

### Local tumor control

We present the largest series comparing the influence of titanium and CFRP spinal hardware in terms of local control after SSRS, finding that 81% of patients were free from local tumor progression after a median follow-up time of 14.5 months. Moulding et al^32^ evaluated local control with high-dose (18-24 Gy) single-fraction SSRS following decompressive surgery and posterolateral instrumentation with both CFRP or titanium implants, finding an overall local control of 81%; this rate increased to 93.8% for cases receiving 24 Gy in a single fraction—however, comparisons between hardware type were not made. Another difference between this series and other reports^33^, ^34^, ^35^, ^36^ is that our cohort had a higher prevalence of renal cell carcinoma (41.0%), which is relatively radioresistant, a property that could have a negative impact on the local control rates. Regarding the specific use of CFRP in combination with SSRS, we report a lower failure rate than other studies that showed progression in 9 of 106 (8.5%) cases of metastatic disease, although only a few of those cases (N = 16) were treated with SSRS.^33^ Shen et al^37^ have found a local recurrence rate of 1 of 7 (14.3%) patients treated with proton therapy for lumbar chordoma. In the retrospective cohort study by Neal et al,^23^ an early local recurrence was found in 3 of 28 cases (10.7%) of spine tumors treated with CFRP implants, with a mean imaging follow-up of 6.5 months, although the radiation modality was not specified. The authors attributed their early detection to the better image quality provided by CFRP. This finding of earlier progression detection was also seen in another study, which demonstrated that progression in CFRP cases was seen 2 times earlier than in patients with titanium, among patients receiving any type of adjuvant radiation, with statistical significance.^38^ Alvarez-Breckenridge et al^11^ investigated 47 metastatic cases, with 24 treated with SSRS, reporting 7 (14.9%) local recurrences, although it was unclear how many had received SSRS. Joerger et al^36^ analyzed a large cohort of 321 patients receiving CFRP instrumentation for primary and metastatic spine disease. They found that 9 of 306 metastatic cases required reoperation to address tumor recurrence when patients were followed for an average of 79 days. The authors did not specify the radiation modality for 258 of 321 (80.4%) patients who received radiation therapy.^36^ A recent, large cohort study conducted by Ward et al^38^ evaluated local oncological outcomes in patients receiving either CFRP or titanium instrumentation followed by radiation (in a total of 148 patients, 45/99 cases in the CFRP group and 15/49 cases in titanium group received SSRS), and found no difference in local control between groups; however, data on the analysis of only patients receiving SSRS were not presented.

Despite the survival analyses demonstrating a significant benefit with the use of CFRP in progression-free survival in our study, this finding was not corroborated by the univariate and multivariate analyses, which showed that only a higher EQD2 (a known independent risk factor for local control failure)^39^ correlated with lower local progression when controlling for multiple variables. The discrepancy in results between different tests may relate to the limited number of patients. The reasons for the difference in dosing schemes were not explored in this study, although it may be related to individual management preferences among the radiation oncologists. Although superior postoperative imaging quality in the CFRP group may facilitate SSRS planning, its actual benefit was likely diluted because SSRS planning often relies on preoperative (preinstrumentation) MRIs, as per our institutional protocol. Although the role of systemic therapy was not addressed in this study, the nonsignificant difference in overall survival between groups suggests comparable systemic tumor control. The relationship between the site of local progression and the location of the spinal implant, particularly with photon radiation, remains unclear. Although the treatment and dosimetric plans account for the density and contour of the metallic implants to deliver a consistent and homogeneous radiation distribution, the higher incidence of in-field failures suggests that cold spots could occur near the metallic implants. We plan to conduct a detailed analysis of this correlation in a follow-up study.

### Hardware failure

We have found similar construct lengths to the ones reported by Joerger et al,^36^ although we have used cement augmentation and vertebral body reconstruction in more cases. These authors identified 55 of 321 cases (17.1%) requiring revision surgery for postoperative complications, with 9 of 321 (2.8%) related to hardware failure: 7 cases of screw loosening, 1 case of titanium rod breakage, and 1 screw fracture. Data on hardware-related complications not requiring surgery were not reported.^36^ In a retrospective cohort study with 28 participants with CFRP spine implants for degenerative lumbar spine disease, Ghermandi et al^40^ found no cases of hardware failure in 1 1-year follow-up.^40^ Only one CFRP case (from 99; 1.0%) presented with hardware failure in a series of oncological patients treated with adjuvant radiation, with no significant difference when compared to a titanium cohort (0/49 cases).^38^ However, a systematic review including 11 studies evaluating CFRP versus titanium construct-related complications in patients with primary and metastatic disease found slightly higher hardware-related complications in the CFRP group.^33^ They identified 21 cases of hardware failure among 316 patients (6.6%), including 10 cases of screw loosening and 5 cases of screw breakage. Twelve cases (5.6%) required reoperation, with 2.8% because of hardware failure.^33^ Although we have not evaluated incidence of intraoperative hardware-related complications, other studies that have done so reported a low risk primarily involving CFRP screw breakages.^36^

This research is the first to compare the clinical outcomes of CFRP versus titanium implants in patients with metastatic spine disease who underwent surgery for spinal cord decompression and posterior segmental stabilization, followed by adjuvant SSRS. We evaluated only constructs made of the same material (either titanium or CFRP). We acknowledge that the CFRP screws used in this study are hybrid, incorporating metallic tulips and set screws. A prior study comparing this specific screw design to an all-titanium system demonstrated a significant reduction in imaging artifacts and no interference with visualization of the spinal canal, which is an important consideration in SSRS planning, as it directly impacts dose distribution to the epidural space and GTV.^9^ We hypothesize that a purely CFRP construct could maximize the radiological and radiation advantages of the material while preserving the biomechanical strength of the implants, though such a comparison is yet to be reported. Our findings suggest that the biomechanical strength of CFRP reconstructions is comparable to that of titanium constructs, with similar rates of hardware failure. We believe the 2 instances of screw breakage requiring revision surgery in our study are biomechanically comparable, and we advise caution when performing short-segment stabilizations, as this appears to be a possible risk factor in our series.

### Strengths and limitations

This study has several limitations associated with its retrospective design. Also, the small sample size may have influenced the discrepancies between the different statistical analyses regarding local progression. Although the Kaplan-Meier and competing risk analyses suggested a protective effect of CFRP, the Cox regression model did not find it significant—this sample size limitation could have reduced the statistical power of the Cox regression, making it less sensitive to detecting an association. The small number of local progression events, particularly in the CFRP group, may have further impacted the reliability of the findings. Larger studies are needed to confirm these results.

The lack of a standardized surgical and radiation treatment protocol may affect the validity of our findings. Notably, patients began receiving CFRP implants only in the past few years (with the oldest CFRP implant dating back to 2019), whereas titanium implants have been used since 2007. This timeframe difference introduces uncontrolled confounders, such as advancements in targeted therapies and improvements in the SSRS technique, including software and hardware development. However, the utilization of preoperative imaging, postoperative CT myelograms, contouring guidelines, spinal cord radiation constraints, minimal dose delivered to the CTV and GTV, remained consistent during the time interval of our study. By narrowing the inclusion criteria to SSRS delivered within 60 days postsurgery, we aimed to reduce confounding factors, albeit at the expense of generalizability. Even considering the time bias, this population represents a real-world cohort often seen in clinical practice at tertiary care centers with advanced multidisciplinary subspecialization. The use of radiology reports to identify local progression can be influenced by interobserver variability and wording differences. We tried to reduce these problems by defining the criteria for progression and having equivocal cases reviewed by an experienced neuro-radiologist and senior neurosurgeons.

It is important to highlight limitations related to the use of CFRP hardware, which may hinder its broad adoption despite the increasing evidence of its benefits. In particular, the cost of these implants is considerably higher than standard titanium hardware. The scarcity of data demonstrating the clinical advantage of the use of CFRP over titanium in the treatment of spinal tumors limits the evaluation of a favorable cost-benefit analysis, because the impact on local control, durability of spine reconstruction, and early detection and treatment of recurrences is yet unknown. The cervical spine was not evaluated because of the lack of available CFRP implants for this segment. Lastly, differences in surgical technique, haptic feedback during screw insertion, and incapacity to bend rods when compared to titanium requires familiarization from the surgeon and team.^11^

## Conclusions

We demonstrated for the first time that utilization of CFRP implants may be associated with superior local progression-free survival than titanium in patients treated with separation surgery followed by SSRS, although this benefit was not seen in the univariate and multivariate analyses—only higher EQD2 was found to be an independent associated factor. The retrospective nature and the small sample of our population limit the interpretation of our results. We speculate that the radiolucent properties of CFRP may enable a more uniform dose distribution within the radiation field than that of titanium. Additionally, we have shown that CFRP constructs provide durable spinal reconstruction in this population, similar to titanium. Detailed radiation phantom studies and a larger cohort study (followed by a randomized prospective study) will be needed to address the possible clinical benefits detected in this study.

## Disclosures

Amol J. Ghia reports speakership/honoraria from icotec. Laurence Rhines is a consultant for icotec and Stryker. Robert Y. North received research support from Stryker. Claudio E. Tatsui received research support from icotec medical. The remaining authors do not have any relevant financial disclosures to report.

No AI has been used in the creation of this article.
